# Prevalence of Overweight in High School Students with Special Reference to Cardiovascular Efficiency

**DOI:** 10.5539/gjhs.v4n2p147

**Published:** 2012-03-01

**Authors:** Aniruddha Deoke, Shilpa Hajare, Ajeet Saoji

**Affiliations:** Department of Community Medicine NKP Salve Institute of Medical Sciences and Research Center, Nagpur, India Tel: 942-215-5266 E-mail: a_rdeoke@rediffmail.com; Department of Community Medicine NKP Salve Institute of Medical Sciences and Research Center, Nagpur, India E-mail: drshilpah@yahoo.com; Department of Community Medicine NKP Salve Institute of Medical Sciences and Research Center, Nagpur, India E-mail: ajeet.saoji@rediffmail.com

**Keywords:** Overweight, students, BMI, Harvard step test, Height, Weight

## Abstract

**Background:**

In India, malnutrition has two ends. Under nutrition attracted the focus of health workers, as it is more prevalent. But over the past few years, childhood obesity is increasingly being observed with the changing lifestyle of families with increased purchasing power, increasing hours of inactivity due to television, video games and computers have replaced outdoor games and other social activities. Globally, an estimated 10% of school-aged children, between 5 and 17 years of age, are overweight and obese. Obesity can be seen as first wave of a defined cluster of non-communicable diseases called “New World Syndrome” creating an enormous socio-economic and public health burden in poorer countries. The most important consequence of childhood obesity is its persistence into adulthood with all its health risks like dyslipidemia, hyper-insulinemia, cardiovascular diseases, type 2 diabetes, osteoarthritis, gall bladder disease, hypertension and some sex hormone- sensitive cancers. The present study was, therefore, undertaken to determine the prevalence of overweight in high school students and to study the association between Body Mass Index (BMI) and cardiovascular efficiency.

**Methodology:**

A school based cross sectional study was conducted in 2 schools of Nagpur. The total number of students included in the study was 565. Student’s complete information regarding profile was taken in pretested questionnaire format after taking informed consent of parents. The anthropometric measurements of the students were done. Student’s height and weight were measured and BMI was calculated. The student’s cardiovascular efficiency was assessed with the help of Harvard step test.

**Statistical Analysis:**

The data was analyzed using Epi info version 3.4.1 software. Chi-square test was used as test of significance and p value less than 0.05 was considered as significant.

**Results:**

90.97% students belonged to 13, 14 and 15 years of age group. majority of the students belong to 14 years (33.81), followed by 13 years (33.27) and 15 years (23.89) of age group respectively. The prevalence of overweight in students was 5.84% and obesity was 0.35%. The combined prevalence of overweight and obesity was 6.19%. The prevalence of overweight in boys is 5.31% and obesity was 0.63% and that in girls is 6.53% and 0% respectively. The association between Harvard step test and overweight was found to be statistically significant (p< 0.000001).

**Conclusion:**

The total prevalence of overweight and obesity was 6.19%. The association between Harvard step test and overweight was found to be statistically significant (p< 0.000001).

## 1. Introduction

Since ancient times of human history, weight gain and fat storage have been viewed as signs of health and prosperity. In times of hard labour and frequent food shortages, securing an adequate energy intake to meet requirements had been the major nutritional concern (WHO, 2000). But now in the present era as standards of living continue to rise and with a drastic change in lifestyle of every individual, weight gain and obesity are posing a growing threat to health in countries all over the world (Mehta M., *et al.*, 2007). Indeed, it is now so common that it is replacing the more traditional public health concerns, including undernutrition and infectious disease, as one of the most significant contributors to ill health (WHO 2000). It can be seen as first wave of a defined cluster of non-communicable diseases called “New World Syndrome” creating an enormous socio-economic and public health burden in poorer countries (Bharti D.R., *et al.*, 2008).

The obesity has been defined as a condition of abnormal or excessive fat accumulation in adipose tissue, to the extent that health may be impaired (WHO 2000).

The development of obesity has been described by Dietz in his article to be linked to four critical or sensitive periods; intra-uterine life, infancy, the period of adipose rebound (5±7 y), and adolescence. Moreover, the onset of obesity during these periods appears to increase the risk of persistent obesity and its complications and has been considered to be an important predictor of long-term morbidity and mortality (Dietz W.H., 1994, Cameron N., *et al.*, 1997). Adolescence seems to be one of the critical periods in the development of obesity. Children belonging to high schools/ senior secondary classes are particularly vulnerable to external factors owing to new found independence and the influence through peer pressure and exposure to media (Aggarwal T. *et al.*, 2008).

Excessive body fat and hypertension are known major risk factors associated with coronary heart disease (CHD), a major cause of morbidity and mortality in many parts of world. Unless controlled early in life, these risk factors tend to impose a higher risk in adulthood. In children and adolescents the rate of increase in body mass index (BMI), but not in height, is a strong predictor of adult levels of blood pressure (BP), insulin resistance and lipids (Mohan B. *et al.*, 2004).

The most important consequence of childhood obesity is its persistence into adulthood with all its health risks. The health risks include dyslipidemia, hyper-insulinemia, cardiovascular diseases, type 2 diabetes, osteoarthritis, gall bladder disease, hypertension and some sex hormone- sensitive cancers (Bharti DR *et al.*, 2008; Laxmaiah A. *et al.*, 2007).

WHO has emphasized on urgent need of understanding the prevalence trend, factors contributing and developing strategies for effective intervention (Kumar S. *et al.*, 2007).

The present study was, therefore, undertaken to determine the prevalence of overweight in high school students and to study the association between Body Mass Index (BMI) and cardiovascular efficiency.

## 2. Materials and Methods

The proposed cross-sectional study was conducted in the high schools of urban area. The total number of schools in urban area was 424. The urban area was divided in 6 zones i.e. North, South, East, West, Central and South-west. Out of the 6 zones one was selected randomly i.e. the south-west zone. The total number of schools in South-west zone was 63. Out of these schools 2 schools were selected again by simple random sampling. All the high school students i.e. students of 8^th^, 9^th^ and 10^th^ class from these 2 schools were included in the study. An informed consent was taken from the Principals of the schools and the parents of the students to carry out the study.

The total number of students in both the high schools were 583 out of which one girl was having severe anaemia and was physically unfit, 12 students’ parents did not give consent and 5 students had left the school. So these students were excluded from the study. The total number of students included in the study was 565. Student’s complete information regarding profile was taken in pretested questionnaire format.

The anthropometric measurements of the students were done i.e. height and weight of the students was measured.

Body Mass Index (BMI): It was calculated with the following formula

BMI = Weight (kg)/ Height (m^2^)

BMI for students was calculated according to WHO 2007 criteria.

The student was considered overweight if his/her BMI was ≥85th percentile and obese if BMI ≥97^th^ percentile according to WHO 2007 sex specific BMI for age percentile charts.

Harvard Step Test: The student was asked to lie down or sit comfortably and his/her basal pulse, blood pressure and respiratory was measured. The student was asked to perform exercise of ascending a platform of 18 inches height 30 times per minute for 5 minutes. If unable to continue exercise for 5 minutes, the time to nearest of second for which he/she exercised was recorded. The student was resumed to his/her original position.

Then his/her pulse was counted between 1 and 1, 1/2 min, 2 and 2, 1/2 min and 3 and 3, 1/2 min. (wait for one minute count for ½ min. wait for ½ min. count for ½ min. wait for ½ min. count for ½ min.). Count the total three readings is called recovery pulse count. The Harvard Index was calculated by following formula-

Harvard Index = (Period of exercise in seconds X 100) / 2 (Recovery pulse count)

Interpretation of score: Above 90- excellent, 80-90 good, 55 -79 average and below 55 poor.

Statistical Analysis: The data was analyzed using Epi info version 3.4.1 software. Chi-square test was used as test of significance and p value less than 0.05 was considered as significant.

## 3. Results

565 students were examined in the study. 90.97% students belonged to 13, 14 and 15 years of age group. majority of the students belong to 14 years (33.81), followed by 13 years (33.27) and 15 years (23.89) of age group respectively. Total number of girls was 43.36% and boys were 56.64%. Of the 565 students’, 367 (64.96%) had normal BMI. The number of students belonging to underweight category constituted 163 (28.85%), whereas 35 students (6.19%) belonged to overweight category. The total prevalence of overweight and obesity was 6.19%. Only 2 students were observed to be obese. Thus the percentage of overweight students was 5.84 and obese students were 0.35.

It was observed that maximum number of students i.e. 66.37% had ’average’ score for Harvard step test and 18.05% had ’poor’ score. Harvard Step Test indicates cardiovascular efficiency. In the analysis of scores of Harvard Step Test it was found that 28 out of 35 overweight students i.e. 80% performed poorly in the test while 7 students i.e. 20% had average performance. None of the overweight students had good or excellent score in the test. The association between Harvard step test and overweight was statistically highly significant (p=0.000001) with odds ratio of 24.65 (C.I. = 9.97; 68.68).

## 4. Discussion

The study revealed that total prevalence of overweight was 5.84% and obesity was 0.35% a total of 6.19% which was similar to other studies conducted in India where the prevalence of overweight ranged from 3.1% - 6.5% and obesity ranged from 0.1%- 1.2% (Supreet Kaur *et al.*, 2008, Bharati *et al.*, 2008). While the studies conducted in bigger cities like Delhi, Hyderabad, Amritsar etc. showed increased prevalence of overweight and obesity ranging from 6.1%-15.2% and 1%- 6.31% respectively (Laxmaiah *et al.*, 2007, M. Shashidhar Kotian *et al.*, 2010, Sharda Sidhu *et al.*, 2005, Bishav Mohan *et al.*, 2004, M. Mehta *et al.*, 2007). The above inferences show that there is higher percentage of obesity in bigger and metropolitan cities of the country. This may be because of increased purchasing power of parents in bigger cities as well as increased frequency of visiting outdoor eating joints and shopping malls. Another reason may be higher standards of living.

In other countries like Australia, Brazil, Saudi Arabia etc. the prevalence of overweight and obesity ranged from 11.9%-23.1% and 2.6%- 9.3% respectively (Pelegrini A *et al.*, 2008, Magarey AM *et al.*, 2001, El Mouzan MI *et al.*, 2010, Del Ri´O-Navarro, BE. *et al.*, 2004) which is much higher than present study findings. This may be because of higher living standards resulting in better accessibility to gadgets and things of comfort. Another reason may be the racial and ethnic difference between India and other countries.

Harvard Step Test indicates cardiovascular efficiency. In the analysis of scores of Harvard Step Test it was found that 28 out of 35 overweight students i.e. 80% performed poorly in the test while 7 students i.e. 20% had average performance. None of the overweight students had good or excellent score in the test. The association between Harvard step test and overweight was statistically highly significant (p=0.000001) with odds ratio of 24.65 (C.l. = 9.97; 68.68). This may be because of load of adipose tissue in overweight students causing overload on pumping mechanism of heart exceeding the anaerobic threshold which in turn causes early fatigue. The findings in other studies were similar to the present study mentioning strong inverse relationship between cardiovascular fitness and excess body weight in adolescents of this age group (M. Fogelholm, *et al*. (2007), Francisco B. Ortega, *et al*. (2007), Bart Drinkard, *et al*. (2001), Pascal Bovet, *et al*. (2007))

## 5. Conclusion

There are many factors which determine the overweight in high school students. In the present study 64.96% students had normal BMI while 6.19% students were overweight. The number of students belonging to underweight category constituted 28.85%.

Out of the 35 overweight students it was found that 19 (5.94%) boys were overweight. Of them 17 (5.31%) boys were overweight and 2 (0.63%) were obese. In the girls 16 (6.53%) were overweight and none were obese.

The students’ cardiovascular efficiency was assessed with the help of Harvard Step Test. The students with ‘excellent’ score were 1.42%, with ‘good’ score were 14.16%, with ‘average’ score were 66.37% and with ‘poor’ score were 18.05%. It was seen that 80% of overweight students had ‘poor’ score in the test while 20% of overweight students had ‘average’ score. The association between Harvard Step Test and overweight was found to be statistically significant (p=0.000001).

Overweight is behavioural problem and it should be tackled by primordial prevention.

As family is the first and most important point of inculcating healthy habits & behaviours, so parents especially mothers as well as children should be made aware about causes and consequences of overweight.

Through extensive BCC activities importance of physical activity, outdoor games, sports and the similar habits should be inculcated in the students and teachers.

Education regarding nutrition, physical activity and hazards of unhealthy lifestyle should be incorporated in the school curriculum.

**Figure 1:**
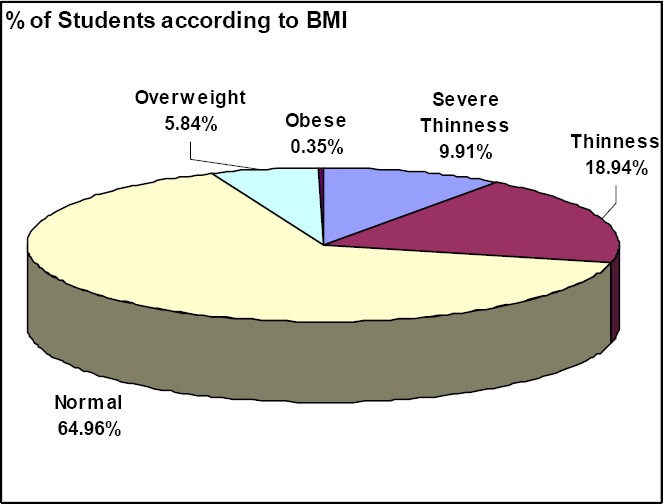
Percentage of Students according to BMI

**Table 1:** Distribution of students according to age and sex (n=565)

**Age** **(years)**	**Sex**		**Total** **(%)**

	**Boys (%)**	**Girls (%)**	
12	7 (53.85)	6 (46.15)	13 (02.30)
13	105 (55.85)	83 (44.15)	188 (33.27)
14	109 (57.07)	82 (42.93)	191 (33.81)
15	73 (54.07)	62 (45.93)	135 (23.89)
16	26 (68.42)	12 (31.58)	38 (06.73)
Total	320 (56.64)	245 (43.36)	565 (100.00)

**Table 2:** Distribution of students according to scores of Harvard Step Test (n=565)

Score	No.	%
Poor	102	18.05
Average	375	66.37
Good	80	14.16
Excellent	8	01.42
Total	565	100.00

**Table 3:** Association between Harvard Step Test and Overweight Students (n=565)

Scores of Harvard Step Test	Students		

	Overweight (%)	Normal (%)	Total
Poor	28(27.45)	74(72.55)	102
Average and above	7(01.51)	456(98.49)	463
Total	35	530	565
